# Rasopathies case report: concurrence of two pathogenic variations de novo in *NF1* and *KRAS* genes in a patient

**DOI:** 10.1186/s12887-019-1463-1

**Published:** 2019-04-05

**Authors:** Irene Baquedano Lobera, Silvia Izquierdo Álvarez, María Jesús Oliván del Cacho

**Affiliations:** 1 Pediatrics Department, Miguel Servet Children’s Hospital, Isabel la Católica Avenue 1-3, 50009 Zaragoza, Spain; 2 Clinical Genetics and Assisted Reproduction, Clinical Biochemistry Department, Miguel Servet Hospital, Padre Arrupe Street, 50009 Zaragoza, Spain; 3 Neonatology Department, Miguel Servet Children’s Hospital, Isabel la Católica Avenue 1-3, 50009 Zaragoza, Spain

**Keywords:** Neurofibromatosis 1, Noonan syndrome, Mutation, Ras-MAPK pathway, Signal transduction pathway, RASopathies, Case report

## Abstract

**Background:**

Rasopathies are a group of genetic malformative syndromes including neurofibromatosis 1, Noonan, LEOPARD, Costello, cardio-facio-cutaneous, Legius, and capillary malformation-arteriovenous malformation syndromes.

**Case presentation:**

We present a female newborn that consulted at the emergency department with refusal to eat and sleepiness. A shortened femur, thickened nucal fold and suspect for agenesis of the corpus callosum were observed in prenatal ultrasound. Her phenotype included hypertelorism, antimongoloid obliquity of the palpebral fissure, prominent forehead, long filtrum, thickened nucal fold, separated nipples, widespread thickened skinfolds and café-au-lait spots. She had a systolic murmur due to pulmonary valve stenosis. The *NF1* gene testing found the pathogenic variant p.E2586X (c.7756G > T) in exon 53, not described in any international database or scientific publications yet. Also, a mutation in the *Kras* gene was detected (p.Val14lle), which is associated with mild Noonan phenotype. Both variations were de novo.

**Conclusions:**

Not all genes and mutations have already been discovered, so it’s important to document new findings, like our patient’s, to enrich and update the international database and broaden all possible knowledge about rasopathies. This is the first case to be described presenting simultaneously two mutations in *Kras* and *NF1* genes, whose possible synergic effect regarding its pathogenicity is unknown, but could be interesting towards therapeutic alternatives.

## Background

Rasopathies are one of the widest groups of genetic malformative syndromes, with an incidence around 1/1000 [1], being the neurofibromatosis type 1 (NF1 [MIM: 162200]) the first one to be identified. Apparently unrelated entities are included such as NF1, Noonan (NS1 [MIM: 163950]), LEOPARD (LPRD1 [MIM: 151100]), Costello (CSTLO [MIM: 218040]), cardio-facio-cutaneous (CFC1 [MIM: 115150]), Legius (MIM: 611431), and capillary malformation-arteriovenous malformation (CMAVM [MIM: 608354]) syndromes.

However, all these syndromes have a common etiopathogenic background, as they develop from germ-line mutations affecting the genes that encode Ras proteins, and the clinical manifestations depend on the protein that is altered in each case.

Ras proteins play an essential role in the cell cycle regulation, growth, differentiation, aging and apoptosis, all of which are critical stages for an appropriate development. Therefore, it is understandable that anomalies in those proteins imply important deleterious effects both in pre and postnatal development. The Ras protein family has been widely studied in cancer and is an attractive therapeutic target for achieving small molecule inhibition with the aim of treating diverse tumoral pathologies [2]. The use of these molecules for improving the developmental defects in rasopathies is being considered.

Ras proteins are codified by the Ras genes, a multigenic family which includes *HRAS, NRAS* and *KRAS*. Ras proteins are monomeric G-proteins with GTPase activity that act as ‘switches’ of the Ras/MAPK pathway, being molecular controllers between effector and receptor proteins in a wide variety of cell signaling pathways, culminating with the activation of the mitogen-activated protein kinase (MAPK).

MAPK is an enzyme with hundreds of substrates that controls cell proliferation, differentiation, migration and apoptosis [3]. Its GTPase activity transforms guanosine triphosphate (GTP) in guanosine diphosphate (GDP) and interrupts the signal, as receptor proteins need GTP to remain activated, becoming inactivated when binded to GDP.

Ras genes host somatic mutations in around 20% of all tumors. However, despite the fact that rasopathies are caused by germ-line mutations in these gene family [3], they also contribute to an activation of oncogenesis, which implies a higher incidence of tumoral diseases in these patients, even though some biochemical studies have shown this activation to be less strong that in the case of somatic mutations.

## Case presentation

We present the case of a 3 year-old patient, who is the first case to be reported associating mutations in *Kras* and *NF1* genes in the same patient, being the *NF1* mutation an undescribed variant until the moment.

She consulted at the emergency department on her ninth day of life due to refusal to eat and sleepiness. No fever or any other infectious signs were present.

She had personal history of shortened femur, thickened nucal fold and suspect for agenesis of the corpus callosum in prenatal ultrasound. Amniocentesis showed a normal fetal caryotype (46, XX). Fetal echocardiography didn’t detect any anomaly and intrauterus fetal magnetic resonance (MRI) was also informed as normal. No intercurrent diseases occurred during pregnancy. She was born by eutocic vaginal delivery with a gestational age of 38 weeks and apgar score 9/10. Her anthropometry at birth was 3590 g of weight (percentile - *p* > 95), 48 cm of leght (p50–75) and 37 cm of cephalic perimeter (p > 95). During the newborn period she was mixed fed (she received both breast and formula milk). The endocrine-metabolic screening had normal results, as well as the otoacoustic emissions.

Physical exploration revealed a facial phenotype with hypertelorism, antimongoloid obliquity of the palpebral fissure, right palpebral ptosis, prominent forehead, and low earlobes. She also had long filtrum, thickened nucal fold, separated nipples, widespread thickened skinfolds and many café au lait spots. She associated a systolic murmur and rhizomelic limbs with stable hips, arched legs and axial hypotonia with normal primitive reflexes.

Regarding her family history, her mother showed an attenuated Noonan phenotype with negative genetic testing and history of feeding difficulties during the newborn period.

During the admission imaging test were performed, giving the transfontanellar and abdominal ultrasound as well as the evaluation of gastrointestinal transit normal results. A normal electroencephalogram was also obtained. The echocardiography showed a moderate pulmonary valve stenosis with a morphologically dysplastic valve. At the age of 14 days of life bone radiological studies were made, including X-ray of the skull, chest, abdomen, spine, limbs and carpi, which were informed as findings suggesting skeletal dysplasia (possible achondroplasia) even though not all the radiological findings fit that entity. Macrocephaly with a prominent frontal bone was observed, as well as short metacarpals, rhizomelic shortness of lower limbs with round enlarged metaphysis, arched legs and delayed appearance of the ossification nucleus.

During the admission she presented progressive feeding difficulties with frequent vomiting and weight loss that prevented from recovering the birth weight, so hypercaloric formula was used with subsequent weight gain. When she was 21 days-old brainstem auditory evoked response (BAER) were practised and showed a mild-to-moderate increased latency of response in wave V with all stimulus intensities, more significant on the left side, suggesting bilateral transmission hearing loss.

In view of the clinical suspicion of Noonan Syndrome, point mutations in the PTPN11, SOS1, RAF1, BRAF1, NF1 and KRAS genes were studied by Sanger sequencing. ADN of peripheral blood was extracted in an automated Maxwell® 16 System, using the Maxwell® 16 LEV Blood DNA kit (Promega). The DNA was quantified by spectrophotometry, in a Biophotometer, through the measurement of the absorbance at a length of 260 nm wavelength. All the coding exons and adjacent intron regions of the 5 genes and the sequencing of the two strands of the amplified fragments and visualization of the sequences by capillary electrophoresis in an Applied Biosystems® 3500DX Genetic Analyzer were amplified by PCR. The sequences obtained consensus for the 5 genes (GenBank Accession Number) were compared.

The *NF1* gene analysis found a pathogenic variant in exon 53 (p.E2586X). This variation is not described in any international database or scientific publications yet, but the predictive algorithms using in silico analysis consider it a pathogenic variant.

Also a mutation in the *KRAS* gene was detected (p.Val14lle), which is known to be associated with mild Noonan phenotype. Both pathogenic variations were de novo in our patient as the genetic testing was negative in both parents.

Throughout the evolutive control an important axial hypotonia persist, as well as horizontal nystagmus that emphasizes if cycloplegic administered, right palpebral ptosis, and optic nerve hypoplasia. The patient is actually awaiting a percutaneous pulmonary valvuloplasty, she needs a nasogastric tube for feeding and follows rehabilitation, physiotherapy and logopaedic plans.

## Discussion and conclusions

Not many years ago, each of the syndromes included under the term rasopathies referred to a specific phenotype and manifestations. However, the change of using the term rasopathies remains in encompassing all the entities previously considered independent as an spectrum of manifestations with a common etiopathogenic origin in mutations affecting the genes that encode the Ras proteins.

Therefore, variations in the Ras/MAPK pathway entail many clinical manifestations susceptible to combine and overlap, such as craniofacial dysmorphology, cardiac malformations, skin, musculoskeletal and ocular anomalies, neurocognitive deficits, hypotonia, and a higher risk for developing tumoral pathology.

This wide spectrum of phenotypical variability is due to the number of genes that can be affected and the diversity of the mutations that can happen in each gene. Hundreds of mutations have already been described using genetic and molecular testing [4], and more continue to be found in genome sequencing studies in patients [5].

For example, patients diagnosed with rasopathies frequently associate cardiovascular anomalies, differing their type and incidence according to the place of theRas/MAPK pathway where the variation takes place. Thus, patients diagnosed with Noonan, Costello or cardio-facio-cutaneous syndromes are more likely to develop hypertrophic cardiomyopathy, pulmonary valve stenosis, septal defects and arrhythmia [6].

Neurofibromin 1 is a protein encoded in the *NF1* gene (17q11.2) whose role is to activate the GTPase activity regulating negatively the *RAS* oncogene, and it is the main negative regulator of the Ras/MAPK pathway, and it is found to be mutated in a wide variety of tumoral pathologies [7]. Over 2000 mutations in the *NF1* gene have been described to be involved in the development of NF1.

The case of our patient allows us to describe a new mutation not described in any international database or scientific publications yet. It is the pathogenic variation p.E2586X, caused by a nucleotide change in heterozygosis affecting position 7756 (c.7756G > T) of exon 53 in the *NF1* gene. This variant changes the 2586 codon of *NF1* gene (that encodes for the glutamic acid aminoacid) into a termination codon, which shortens the protein (truncated protein) to 2585 aminoacids, in stead of the 2818 aminoacids that the normal protein has.

Moreover, it is the first case to be described simultaneously associating two pathogenic mutations in *NF1* and *KRAS* genes. There is one documented case with concurrence of two pathogenic mutations in *NF1* and *PTPN11* genes that was published in 2005 [8].

The diagnosis for rasopathies begins with the clinical and phenotypical features recognition during the medical exam, with subsequent genetic testing for confirmation. Nevertheless, not all the genes involved in the development of rasopathies have been identified yet.

It has been observed in tumorigenesis models that anomalies in occurred in *KRAS* genes are meaningfully stronger than those affecting *HRAS*.

The existence of a mutation in *KRAS*, but not in *HRAS* nor *NRAS*, boosts tumor development by inhibiting the calmodulin kinase. Therefore, the interruption of that interaction is an important therapeutic target, having observed that its interruption abolishes tumoral growth in preclinical models.

Regarding to the treatment, on one hand we have to manage the clinical manifestations and complications that appear throughout evolution, such as valvuloplasty for the pulmonary stenosis. On the other hand, there are numerous studies and trials in different phases that test the effect of diverse substances, most of which target the modulation of the Ras/MAPK pathway at different points [9], but there are no conclusive results for the moment.

For example, 3-hydroxy-3-methylglutaryl coenzyme-A reductase inhibitors have been used in clinical trials to reinforce cognitive function in patients diagnosed with NF1 [10].

However, as more refined tools such as MAPK selective inhibitors used to ease neuropathic pain in rats [11], or phenotypic gastrulation in zebrafish models [12] become available, the Ras/MAPK pathway will turn into target of new therapies pursuing to counteract axonal degeneration, enable posttraumatic functional recuperation and restore cognitive function in rasopathies [13].

We highlight once again the importance of the change of direction in approaching genopathies in general, and rasopathies in particular, as the approach based on the genotype to explain the phenotype affects meaningfully the comprehension of the fisiopathology and allows to broaden and revolutionize the choices and therapeutic targets.

## Abbreviations

BAER: Brainstem auditory evoked response
EEG: Electroencephalogram
GDP: Guanosine diphosphate
GTP: Guanosine triphosphate
MAPK: Mitogen-activated protein kinase
MRI: Magnetic resonance imaging
NF1: Neurofibromatosis type 1
*NF1*: Neurofibromin 1 gene
P: Percentile
